# Placental inflammation and overweight or obesity in term singleton stillbirths in Stockholm County 2002–2018; a case control study

**DOI:** 10.1371/journal.pone.0284525

**Published:** 2023-04-18

**Authors:** Hanna Åmark, Lottie Säker, Nikos Papadogianakis

**Affiliations:** 1 Department of Clinical Science and Education, Unit of Obstetrics and Gynecology, Karolinska Institute, Södersjukhuset, Stockholm, Sweden; 2 Department of Gynecology and Obstetrics, Södersjukhuset, Stockholm, Sweden; 3 Dep of Laboratory Medicine, Division of Pathology, Karolinska University Hospital Huddinge and Karolinska Institutet, Solna, Sweden; University of Palermo: Universita degli Studi di Palermo, ITALY

## Abstract

**Introduction:**

Stillbirth is a severe pregnancy complication. Maternal obesity is one of the most important modifiable risk factors of stillbirth, yet the biological mechanisms behind this association remain unclear. The adipose tissue is an endocrine organ which, in persons with obesity, causes a hyperinflammatory state. The aim of this study was to investigate inflammation as a contributing mechanism to the risk of stillbirth in women with obesity and if there are possible signs of different BMI phenotypes with different risk.

**Material and methods:**

This was a case control study based on all cases of term singleton stillbirth without major fetal malformation in Stockholm County between 2002–2018. Placentas have been examined according to a standardized protocol. Placental inflammatory lesions were compared both between placentas from pregnancies with live born and stillborn infants with different class of body mass index (BMI) as well as among women with stillborn and live born infants with different classes of BMI, respectively.

**Results:**

All inflammatory placental lesions were more common in placentas from women with stillbirth compared to placentas from women with live born infants. Vasculitis, funisitis and chronic villitis as well as overall fetal and maternal inflammatory response were present with a significantly increased proportion with increasing BMI in placentas from women with term stillbirth however, there were no differences between placentas from women in different BMI classes with term live born infants.

**Conclusion:**

Both acute and chronic inflammatory placental lesions were more common in cases of stillbirth compared to pregnancies with live born infants. There were increased proportions of both acute and chronic placental inflammation (vasculitis, chronic villitis, funisitis and overall fetal and maternal inflammatory response) with increasing BMI among cases with term stillbirth, however no differences among controls with term live born infants.

## Introduction

Obesity is an increasing worldwide pandemic [1]. Increasing body mass index (BMI) is associated with risks during pregnancy, labor and the postpartum period [2]. It is well known that the risk of stillbirth increases with increasing BMI [3–7]. The increased risk of stillbirth associated with BMI is most prominent among term pregnancies [3]. Obesity is the most important modifiable risk factor for stillbirth [7].

The reasons behind the increased risk of stillbirth in women with overweight or obesity are not fully understood, however there are probably several different contributing reasons [6]. There is an association between BMI and stillbirth caused by placental disease, maternal hypertension, umbilical cord complications and antepartum infections [5, 6, 8]. An altered metabolism with potentially elevated risk of fetal hypoxia may contribute, as well as an inflammatory process [5, 9]. The adipose tissue is an endocrine organ with production of pro-inflammatory cytokines [10]. It is shown that obese pregnant women have an enhanced inflammatory state which seems to affect the placental inflammatory state [11]. There is research indicating placental pathology as mediating part of the effect of BMI on stillbirth [5, 8]. The reasons behind the elevated risk of stillbirth with increasing BMI are complex due to different mechanisms, however they may be complex also due to different BMI phenotypes. BMI phenotypes are discussed according to the risk of, for example, cardiovascular events [12].

The aim of this study was to compare frequencies of placental lesions associated with inflammation in pregnancies complicated by term singleton stillbirth compared to uneventful term singleton pregnancies according to maternal pre-pregnancy BMI. Cases and controls will be compared and there will also be comparison between BMI classes among controls and cases, respectively, to be able to evaluate placental inflammation as a contributing mechanism behind risk of stillbirth in obese women as well as the possibility of different BMI phenotypes.

## Material and methods

This is a case control study with cases of term singleton stillbirths in the Stockholm County between 2002 and 2018. The case control design was chosen because of the uncommon outcome. Stillbirth cases were identified from the Stockholm Stillbirth data base, which contains detailed information about 587 stillbirths diagnosed from gestational week (GW) 37+0 during the time period. Virtually all cases of stillbirth in Stockholm County were included. The Stockholm stillbirth database is part of the Swedish Pregnancy Register (Graviditetsregistret.se). Stillbirths with major fetal malformations were excluded. The Stockholm stillbirth group has collected information on each stillbirth in the Stockholm County since 1998 based on the same protocol [13] and with data from antenatal records, blood samples, microbiology tests, chromosomal analyses, placental analyses and fetal autopsy. In a structured, consensus driven process primary and secondary causes of death as well as the degree of certainty of the cause of death have been established [13, 14]. Controls were women with uneventful pregnancies collected between 2002–2005 and 2018–2019, for the purpose of study and not because of suspicious placental pathology. Controls were not matched to cases, however collected consecutively. To be able to evaluate possible effects of different BMI phenotypes comparisons were made both between pregnancies complicated by stillbirth and uneventful pregnancies as well as between different maternal BMI among pregnancies complicated by stillbirth and uneventful pregnancies, respectively.

A systematized examination protocol essentially in agreement with the Amsterdam consensus criteria has been used for the placental analyses, which comprised a two-step macroscopic and histologic evaluation and included standardized sampling, reporting and definition of histopathologic lesions [15]. The examination protocol was established in 2001 and has been described and implemented by our group [9, 14]. Stillbirths before 2002 were excluded since all the placental analyses prior to 2002 were not done according to the examination protocol.

Placental analyses for controls were done according to the same examination protocol. All placentas were sent fresh to the Section for Perinatal Pathology at Karolinska University Hospital, Huddinge. Placentas were analyzed by senior perinatal pathologists. The pathologists were blinded for maternal BMI and other maternal characteristics however, pathologists were not blinded for stillbirth/live birth because cases were collected after clinical cases of stillbirth.

All placentas were analyzed according to a standardized examination protocol including several parameters, however in this paper we focused on signs of inflammation in the placenta. Acute chorioamnionitis was defined histologically as infiltration of polymorphonuclear leukocytes (granulocytes) into the fetal membranes. It was assessed in a two-grade scale, grade 1; involvement of subchoreal fibrin layer or/and chorionic tissue; and grade 2; involvement of amniotic membranes. Vasculitis was defined as granulocytic infiltration of the vessel walls in the chorionic plate or vessels of the umbilical cord. Funisitis was defined as presence of granulocytes in umbilical cord stroma (Wharton’s jelly). Chronic villitis was defined as mononuclear cell infiltration in villous stroma; the presence and extent of chronic villitis were assessed histologically and divided in two categories <1% and >1%. These categories were chosen with respect to clinical relevance. <1% represents a few sporadic foci with 3–10 affected villi and >1% represents the total villous area involved by the inflammatory process and more than 10 villi affected. According to the Amsterdam criteria, we also analyzed fetal inflammatory response, i.e. vasculitis or funisitis as well as maternal and fetal inflammatory response, i.e. both maternal inflammatory response (chorioamnionitis grad 2) and fetal inflammatory response in the same placenta.

The antenatal records were used to collect information on maternal characteristics. BMI was based on self-reported height and measured weight at the first antenatal visit during the first trimester of pregnancy. Parity was handled as a binomial variable, i.e. primipara yes/no. Smoking was recorded as yes/no at the first antenatal visit in the first trimester. Country of birth was handled as a binomial variable i.e. born in Sweden or not.

There was in general a low degree of missing data. There were in total 12 placentas missing. In addition, approximately 1.5% missing data for each inflammatory placental variable.

### Ethical approval

For this study was obtained from the Regional Research Ethics Committee at Karolinska institute in Stockholm, Sweden 2017/14-31/4 approved 9^th^ of March 2017, with amendment 2019–05991, approved 15^th^ of February 2020. According to the ethical approvement anonymized information about women with stillbirths came from the Stockholm Stillbirth database and these women did not give an informed consent. All included women with live born infants gave a written informed consent.

### Statistical analyses

Maternal characteristics were compared between women with term stillbirth and term live born infants according to maternal pre-pregnancy BMI. Comparisons between continuous variables were done with Wilcoxon rank sum test, presented as median and interquartile range and comparisons between categorical variables were done with chi-square test, presented as proportions. Frequencies of placental variables indicating inflammation were compared between placentas from women with term singleton stillbirth and placentas from women with term live born singleton infants according to maternal pre-pregnancy BMI. Frequencies of placental variables indicating inflammation were also compared between placentas from women with different BMI classes and term singleton stillbirth and term singleton live born infants, respectively. The differences in proportion of inflammatory placental lesions over the three BMI classes among pregnancies with stillbirth and live birth, respectively were tested using chi-square test for multiple proportions and showed in figures. Different causes of stillbirth were compared between pregnancies in different BMI classes using chi-square test.

Analyses were done using R crane.

## Result

**Fig 1:**
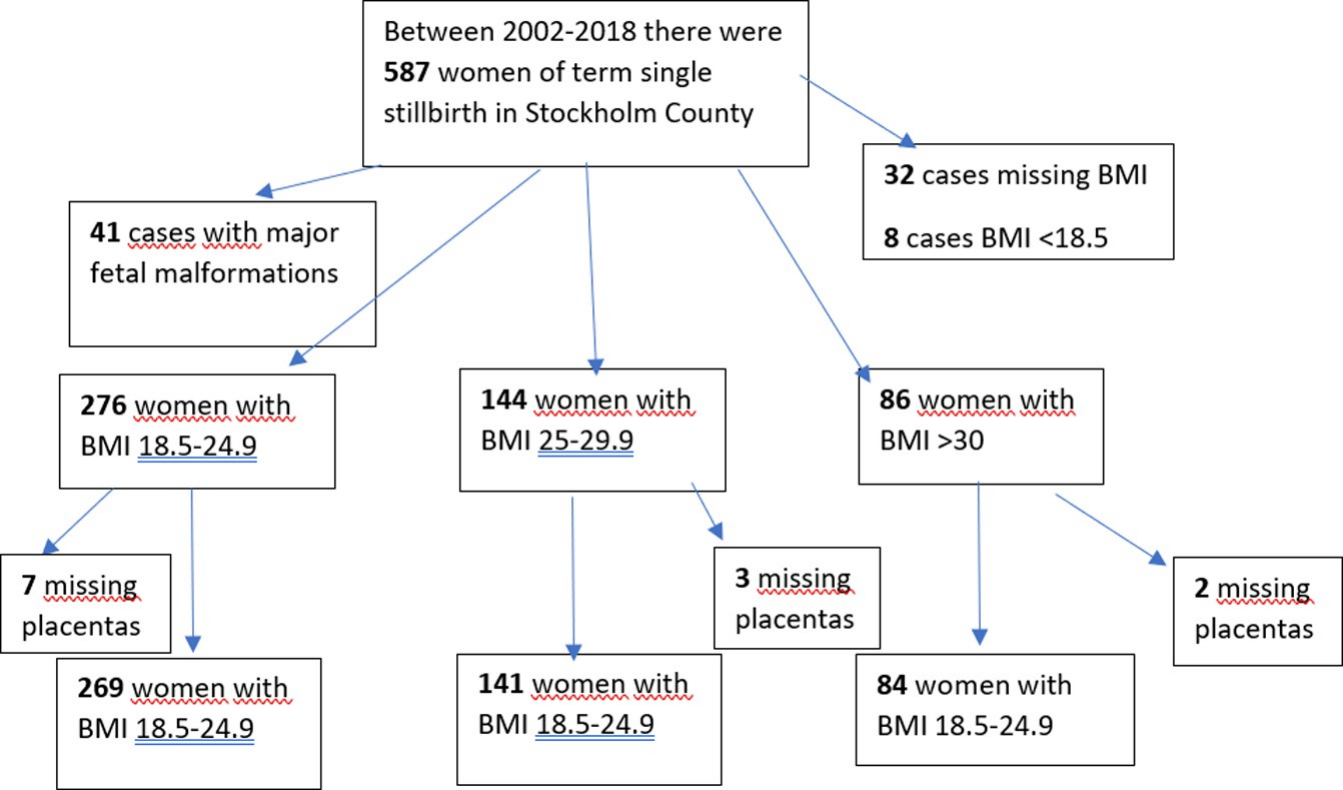
Flow-chart over number of cases.

The Stockholm stillbirth database contains information on 587 term singleton stillbirths born between 2002 and 2018. After exclusion of 41 stillbirths due to major fetal malformations, 32 stillbirths with missing BMI and 8 stillbirths with BMI<18.5, there were 506 cases. In addition, there were 12 (2.4%) stillbirths without placental analyses. There were 7 cases with BMI 18.5–24.9 without placental analyses, 3 cases with BMI 25–29.9 without placental analyses and 2 cases with BMI ≥30 without placental analyses, respectively. Our final cohort included 494 term singleton stillbirth divided in three different BMI classes; n = 269 term stillbirths with women of normal BMI (>18.5 to <25), n = 141 term stillbirth with women with BMI ≥25 to <30 and n = 84 term stillbirth with women with BMI ≥30, Fig 1.

**Fig 2:**
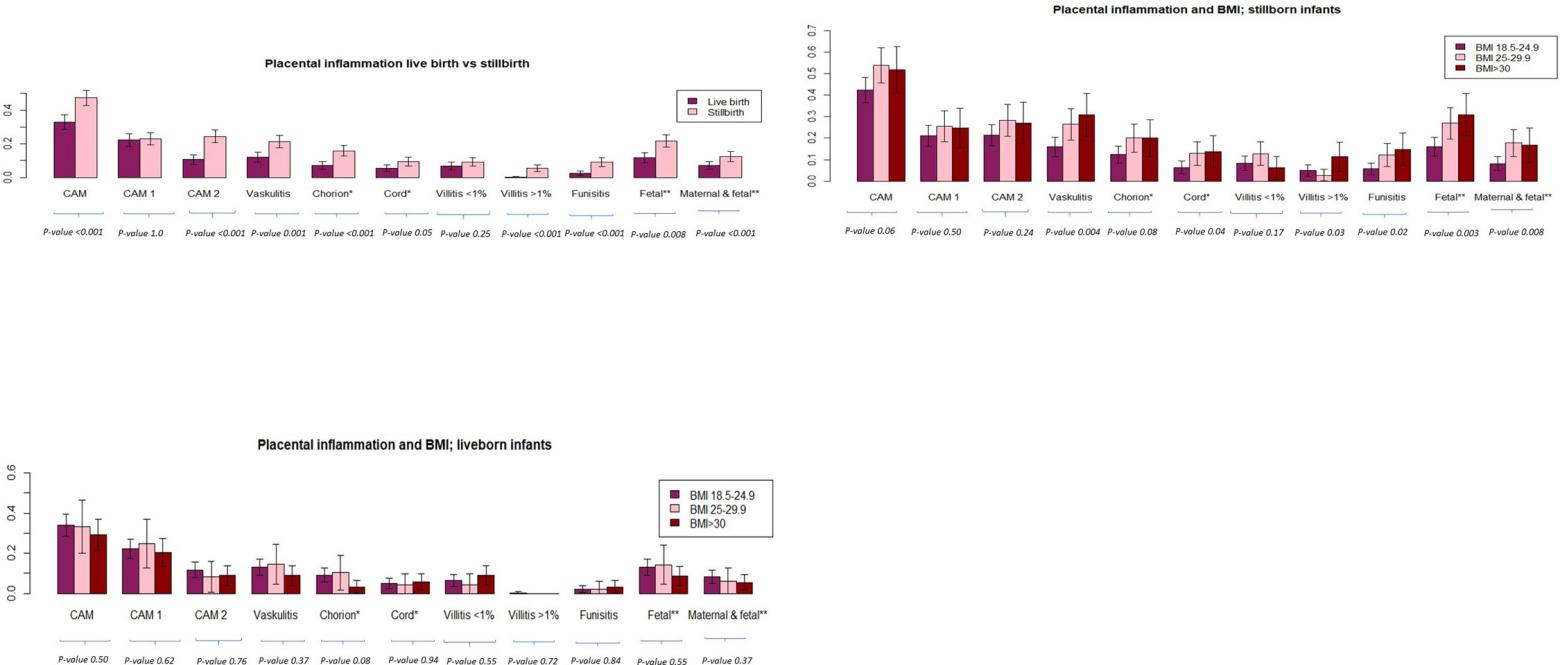
**a.** Placental inflammatory lesions compared between term pregnancies with live born infants and term pregnancies complicated by stillbirth. CAM–chorioamnionitis, *—vasculitis, **—inflammatory response. **b.** Placental inflammatory lesions from term pregnancies complicated by stillbirth, divided by maternal pre-pregnancy BMI. Chi-square test for multiple proportions was used to evaluate significant differences. CAM–chorioamnionitis, *—vasculitis, **—inflammatory response. **c.** Placental inflammatory lesions from term pregnancies with live born infants, divided by maternal pre-pregnancy BMI. Chi-square test for multiple proportions was used to evaluate significant differences. CAM–chorioamnionitis, *—vasculitis, **—inflammatory response.

Maternal characteristics for term, singleton pregnancies with live born infants and stillbirth according to BMI are shown in Table 1. More women with stillbirth were born outside Sweden, for all BMI classes. Among normal weight women there was a lower proportion of nulliparas and a lower gestational age among pregnancies complicated by term stillbirth. Placental lesions indicating both acute and chronic inflammation were found in larger proportions comparing stillbirth and live born infants, as shown in Tables 2 and 3 and Fig 2A. When grading chorioamnionitis and chronic villitis, chorioamnionitis grade 1 and chronic villitis <1% were not found in increased proportions, however chorioamnionitis grade 2 and chronic villitis >1% were found in increased proportions in placentas from women with term stillbirth compared to placentas from women with live born infants. Vasculitis, both in chorionic plate and umbilical cord, chronic villitis >1%, funisitis and fetal inflammatory response as well as maternal and fetal inflammatory response were more frequent with increasing BMI in cases with term singleton stillbirth, shown in Fig 2B. Placental lesions indicating inflammation from placentas of women with live born infants did not differ according to BMI classes, Fig 2C.

**Table 1:** Maternal characteristics comparing term pregnancies with live born infants with term pregnancies complicated by stillbirth, according to pre-pregnancy BMI in Stockholm county 2002–2018. For continuous variables medians with interquartile range are expressed and for categorical variables numbers and proportions are expressed.

Maternal characteristics	Normal weight live born n = 276	Normal weight stillborn n = 269	P-value	Overweight live born n = 49	Overweight stillborn n = 141	P-value	Obese live born n = 129	Obese stillborn n = 84	P-value
Maternal age, years	31.94 (28,35)	32 (29,36)	0.326	31 (28,34)	32 (27,35)	0.741	32.59 (30.08,36.25)	34 (30,36.5)	0.554
BMI, kg/m2	22.05 (20.6,23.4)	22.6 (21.11,23.8)	0.004	26.7 (25.96,27.89)	27.22 (26.08,28.37)	0.278	32.51 (31.24,34.72)	32.01 (30.97,34.37)	0.27
Nullipara (n, %)	151 (54.91%)	105 (45.06%)	0.034	21 (42.86%)	55 (44%)	1	52 (40.31%)	29 (39.19%)	0.994
Smoking (n,%)	12 (4.35%)	18 (8.11%)	0.118	1 (2.04%)	8 (6.78%)	0.391	1 (0.78%)	8 (11.43%)	0.002
Mother born in Sweden(n, %)	208 (82.54%)	153 (69.86%)	0.002	27 (64.29%)	56 (47.46%)	0.09	87 (71.9%)	37 (55.22%)	0.032
Gestational age, days	280 (274,285)	277 (267,284)	<0.001	279 (271,284)	279 (271,286)	0.98	280 (275,287)	280.5 (267.75,286.25)	0.307
Pre-eclampsia (n, %)	3 (1.09%)	1 (0.45%)	0.772	0 (0%)	3 (2.52%)	0.631	2 (1.55%)	7 (10%)	0.017

**Table 2:** Placental inflammatory lesions from term pregnancies with live born infants and stillborn infants. For continuous variables medians with interquartile range are expressed and for categorical variables numbers and proportions are expressed.

Fetal and Placental characteristics	Live born n = 477	Stillborn n = 494	P-value
Fetal weight, g	3515 (3222,3845)	3250 (2920,3663.75)	<0.001
SGA >10th percentile (n, %)	36 (7.55%)	119 (24.29%)	<0.001
LGA >90th percentile (n, %)	58 (12.16%)	46 (9.39%)	0.198
Placental weight, g	492 (426,556)	430 (363,500.75)	<0.001
Placenta hypoplasia (n, %)	61 (12.79%)	246 (49.8%)	<0.001
Placental hyperplasia (n, %)	29 (6.08%)	29 (5.87%)	0.998
Chorioamnionitis(n, %)	154 (32.91%)	231 (47.34%)	<0.001
Chorioamnionitis grade 1(n, %)	104 (22.22%)	112 (22.95%)	0.848
Chorioamnionitis grade 2(n, %)	50 (10.68%)	119 (24.39%)	<0.001
Vaskulitis (n, %)	56 (11.99%)	104 (21.44%)	<0.001
Vaskulitis chorionplate (n,%)	34 (7.28%)	77 (15.81%)	<0.001
Vaskulitis cord (n, %)	25 (5.35%)	46 (9.45%)	0.022
Funisitis (n, %)	12 (2.57%)	44 (9.07%)	<0.001
Chronic villitis (n, %)	33 (7.07%)	71 (14.64%)	<0.001
Chronic villitis<1%(n, %)	32 (6.85%)	45 (9.28%)	0.21
Chronic villitis >1% (n, %)	1 (0.21%)	26 (5.36%)	<0.001
Fetal inflammatory response* (n, %)	56 (11.74%)	107 (21.66%)	<0.001
Fetal and Maternal inflammatory response** (n, %)	34 (7.13%)	61 (12.35%)	0.009

* vasculitis or funisitis,

** fetal inflammatory response and chorioamnionitis grade 2.

**Table 3:** Placental inflammatory lesions from term pregnancies with live born infants and stillborn infants according to pre-pregnancy maternal BMI. For continuous variables medians with interquartile range are expressed and for categorical variables numbers and proportions are expressed.

Fetal and Placental Characteristics	Normal weight live born n = 276	Normal weight stillborn n = 269	P-value	Overweight live born n = 49	Overweight stillborn n = 141	P-value	Obese live born n = 129	Obese stillborn n = 84	P-value
Fetal weight, g	3492.5 (3200,3820)	3220 (2970,3600)	<0.001	3480 (3230,3830)	3250 (2848.75,3803.75)	0.017	3610 (3290,3920)	3478 (3015,3723.75)	0.002
SGA >10th percentile (n, %)	23 (8.33%)	63 (23.68%)	<0.001	4 (8.16%)	38 (27.14%)	0.011	7 (5.43%)	18 (21.43%)	<0.001
LGA >90th percentile (n, %)	32 (11.59%)	19 (7.14%)	0.104	3 (6.12%)	18 (12.86%)	0.304	20 (15.5%)	9 (10.71%)	0.429
Placental weight, g	487 (418.25,546)	427 (364,487.5)	<0.001	500.5 (450.5,563.25)	428 (354,512.5)	<0.001	504.5 (443.75,580)	455 (381,533)	<0.001
Placenta hypoplasia (n, %)	36 (13.04%)	142 (52.79%)	<0.001	1 (2.04%)	65 (46.1%)	<0.001	24 (18.6%)	39 (46.43%)	<0.001
Placental hyperplasia (n, %)	9 (3.26%)	15 (5.58%)	0.268	0 (0%)	12 (8.51%)	0.077	19 (14.73%)	2 (2.38%)	0.007
Chorioamnionitis(n, %)	93 (33.94%)	113 (42.48%)	0.051	16 (33.33%)	76 (53.9%)	0.022	36 (29.27%)	42 (51.85%)	0.002
Chorioamnionitis grade 1(n, %)	61 (22.26%)	56 (21.05%)	0.813	12 (25%)	36 (25.53%)	1	25 (20.33%)	20 (24.69%)	0.573
Chorioamnionitis grade 2(n, %)	32 (11.68%)	57 (21.43%)	0.003	4 (8.33%)	40 (28.37%)	0.008	11 (8.94%)	22 (27.16%)	0.001
Vaskulitis (n, %)	36 (13.19%)	42 (15.91%)	0.44	7 (14.58%)	37 (26.43%)	0.14	11 (8.94%)	25 (30.86%)	<0.001
Vaskulitis chorionplate (n,%)	25 (9.16%)	33 (12.36%)	0.288	5 (10.42%)	28 (20%)	0.198	4 (3.25%)	16 (20%)	<0.001
Vaskulitis cord (n, %)	14 (5.13%)	17 (6.37%)	0.664	2 (4.17%)	18 (12.86%)	0.157	7 (5.69%)	11 (13.75%)	0.085
Funisitis (n, %)	6 (2.19%)	15 (5.68%)	0.062	1 (2.08%)	17 (12.14%)	0.078	4 (3.28%)	12 (14.81%)	0.007
Chronic villitis	19 (6.93%)	35 (13.21%)	0.023	2 (4.17%)	22 (15.6%)	0.071	11 (9.02%)	14 (17.72%)	0.108
Chronic villitis<1%(n, %)	18 (6.57%)	22 (8.3%)	0.547	2 (4.17%)	18 (12.77%)	0.161	11 (9.02%)	5 (6.33%)	0.674
Chronic villitis >1% (n, %)	1 (0.36%)	13 (4.91%)	0.002	0 (0%)	4 (2.84%)	0.549	0 (0%)	9 (11.39%)	<0.001
Fetal inflammatory response* (n, %)	36 (13.04%)	43 (15.99%)	0.393	7 (14.29%)	38 (26.95%)	0.109	11 (8.53%)	26 (30.95%)	<0.001
Fetal and Maternal inflammatory response** (n, %)	23 (8.33%)	22 (8.18%)	1	3 (6.12%)	25 (17.73%)	0.082	7 (5.43%)	14 (16.67%)	0.014

* vasculitis or funisitis,

** fetal inflammatory response and chorioamnionitis grade 2.

There was no difference in cause of stillbirth between groups, Table 4.

**Table 4:** Cause of fetal death in term pregnancies complicated by stillbirth in Stockholm county 2002–2018, divided according to maternal pre-pregnancy BMI.

Cause of fetal death	BMI 18.5–24.9 n = 269	BMI 25–29.9 n = 141	P-value	BMI≥30 n = 84	P-value
Infection	59 (23.41%)	41 (32.28%)	0.084	23 (31.08%)	0.984
Feto-maternal transfusion	10 (3.97%)	5 (3.94%)	1	0 (0%)	0.208
Placental insufficiency/IUGR	93 (36.9%)	45 (35.43%)	0.867	25 (33.78%)	0.934
Umbilical cord complications	27 (10.71%)	10 (7.87%)	0.486	3 (4.05%)	0.444
Placental abruptio	22 (8.73%)	8 (6.3%)	0.531	7 (9.46%)	0.586
Preeclampsia	1 (0.4%)	0 (0%)	1	3 (4.05%)	0.092
Diabetes mellitus	5 (1.98%)	7 (5.51%)	0.123	1 (1.35%)	0.28
Cause of stillbirth un-known	23 (9.13%)	11 (8.66%)	1	10 (13.51%)	0.398

## Discussion

Placental lesions indicating inflammation were more frequent in cases of term singleton stillbirth compared to controls with term live born infants when all classes of BMI were included. Frequencies of placental lesions indicating both acute and chronic inflammation, i.e; vasculitis, funisitis and chronic villitis, increased with increasing BMI in pregnancies complicated by term stillbirth. There were no differences in placental lesions indicating inflammation when comparing placentas from uneventful pregnancies with different BMI classes with live born infants.

A placental inflammatory process contributes to the risk of stillbirth and that contribution is potentially more pronounced in women with overweight or obesity. It is well known that placental inflammatory lesions are more frequent in placentas from women with stillbirth [16, 17]. The indication that the inflammatory process have a larger contribution to the risk of stillbirth among women with overweight or obesity is supported by previous studies [5, 6]. The fact that there were no differences in placental lesions among uneventful pregnancies with live born infant may indicate that women with obesity and stillbirth are different from women with obesity and live born infants. Those differences may impact on the risk of stillbirth and are possibly associated to the reasons behind the increased risk.

Among non-pregnant persons there are different obesity phenotypes with for example different fat mass distribution and according to that different risk of, for example, heart failure even with equivalent BMI [12]. There may be different obesity phenotypes also among pregnant women, with different levels of inflammation and/or different possibilities to handle the inflammatory process. Different phenotypes could potentially explain that there were no differences between placentas from uneventful pregnancies with different BMI classes, although there were differences with increasing BMI among cases with stillbirth.

Chorioamnionitis represents an acute inflammatory reaction and is a common placental lesion also in uncomplicated pregnancies [18, 19]. The prevalence of chorioamnionitis in placenta increases with spontaneous labor, premature labor or ruptured membranes [18]. Low-grade chorioamnionitis is an intriguing lesion. Traditionally, it has been regarded as the early stage in the temporal evolution of chorioamnionitis (an ascending bacterial infection), representing the initial, purely maternal response [18]. However, some previous results indicate that it may, at least partly, reflect a normal inflammatory process associated with labor [19]. There were no differences in proportion of chorioamnionitis grade 1 when comparing live born and stillborn infants or when comparing women with normal weight and women with obesity, supporting the theory of chorioamnionitis grade 1 reflecting a normal inflammatory process associated with labor.

Chorioamnionitis grade 2 was also a common placental lesion, however more common in placentas from cases of term stillbirth. Vasculitis and funisitis are generally believed to represent signs of fetal inflammatory response [21]. Previous research has shown an association between stillbirth and acute and chronic vasculitis [20]. Vasculitis and funisitis were more common with increasing BMI in pregnancies complicated by stillbirth, supported by a study investigating inflammation and inflammatory associated biomarkers associated with stillbirth [5]. However, this study by Harrison et al could not find satisfying predictive biochemical markers for the pathway from obesity via inflammation to stillbirth [5].

Previous studies have shown differences in frequencies of chronic villitis between uncomplicated pregnancies and pregnancies complicated by for example stillbirth, supporting our findings [25]. Chronic villitis is characterized by chronic inflammatory infiltrate with ensuing destruction of villous parenchyma including trophoblastic layers. Thus it may affect the placental barrier and lead to placental dysfunction/insufficiency and subsequent growth restriction or stillbirth, both of which are strongly associated with the presence of chronic villitis [21]. In most cases the etiology of chronic villitis is unknown; immunological mechanisms resembling graft-versus-host reaction are inferred and it has been shown that the majority of invading leukocytes are of maternal origin [22]. An increased level of maternal inflammation could potentially contribute to an increased proportion of chronic villitis with increasing BMI [23].

There are biological mechanisms supporting the possible impact of inflammation as a potential cause of the increased risk of stillbirth in obese women [24]. The adipose tissue is an endocrine organ with an enhanced pro-inflammatory effect in persons with obesity, associated with secretion of proinflammatory cytokines both locally and systematically [23, 24]. The proinflammatory cytokines lead to an ongoing inflammatory process in pregnant women with obesity, believed to contribute to the development of preeclampsia and gestational diabetes [25]. In non-pregnant persons with obesity, the enhanced inflammatory process contributes to the increased risk of cardiac disease [26], with a theoretical potential to affect also placental blood-vessels and their development.

The results indicate that the effect of an inflammatory placental process on term stillbirth is greater with increasing BMI. However, to be able to use that knowledge to decrease the risk in future pregnancies a biomarker would be needed. For example, recent research has shown an association between uterine arteries pulsatility index and placental lesions of maternal vascular malperfusion [27]. Previous research has also shown that changes in miRNA could affect placental function [28]. These are fields of continued research with the aim to find reliable biomarkers.

One strength of this study is the large number of term singleton stillbirths, which is an uncommon outcome. All stillbirths have been evaluated in an audit process and the placentas have been examined by a small number of experienced perinatal pathologists according to a specific, standardized examination protocol. The stringent way of placental examinations and validation of each case of stillbirth based on the same protocol lead to a high degree of replicability and reproducibility.

One limitation of this study is that stillbirth is a rare event, leading to inclusion over a long time period to have an adequate number of cases. During the study period the obstetric care has evolved in several aspects, the criteria for pre-eclampsia and gestational diabetes have been changed [29]. The surveillance of pregnancies has evolved, the possibilities to use ultrasound as a diagnostic tool has evolved, the knowledge about risks has grown and interventions are used in earlier gestational weeks. The changes in the obstetric care are possibly affecting the outcome, the number of stillbirths. In addition, the frequency of the exposure has changed during the study period [30]. Guidelines to decrease the risk of stillbirth in a certain group may affect the causal pathways. Live born infants are used as a comparison to evaluate if the same differences in placental inflammatory lesions are found also in uneventful pregnancies. We note that there is a low number of women with live born fetuses, especially with BMI 25 to 29.9. Another limitation is that the pathologists were blinded for maternal BMI but not for stillbirth versus live birth. This may entail a risk of over-estimation of placental pathologic findings in our material.

## Conclusion

Placental inflammatory processes are more pronounced in cases of term stillbirth compared to term live births. In addition, both vasculitis, funisitis, chronic villitis as well as an overall fetal and maternal inflammatory response were found more frequently in placentas from women with overweight or obesity in pregnancies complicated by term singleton stillbirth. Both acute and chronic inflammation may contribute to the increased risk of stillbirth of women with increased BMI.

## Abbreviations

BMI: body mass index

## References

[1] WHO. Obesity and overweight [Internet]. Geneva: World Health Organization; 2021. Available from: https://www.who.int/news-room/fact-sheets/detail/obesity-and-overweight.

[2] Mission JF, Marshall NE, Caughey AB. Pregnancy risks associated with obesity. Obstet Gynecol Clin North Am. 2015;42(2):335–53. Epub 2015/05/24. doi: 10.1016/j.ogc.2015.01.008 . 26002170

[3] Yao R, Schuh BL, Caughey AB. The risk of perinatal mortality with each week of expectant management in obese pregnancies. The journal of maternal-fetal & neonatal medicine: the official journal of the European Association of Perinatal Medicine, the Federation of Asia and Oceania Perinatal Societies, the International Society of Perinatal Obstet. 2017:1–8. Epub 2017/09/20. doi: 10.1080/14767058.2017.1381903 . 28922969

[4] Aune D, Saugstad OD, Henriksen T, Tonstad S. Maternal body mass index and the risk of fetal death, stillbirth, and infant death: a systematic review and meta-analysis. Jama. 2014;311(15):1536–46. Epub 2014/04/17. doi: 10.1001/jama.2014.2269 . 24737366

[5] Harrison MS, Thorsten VR, Dudley DJ, Parker CB, Koch MA, Hogue CJR, et al. Stillbirth, Inflammatory Markers, and Obesity: Results from the Stillbirth Collaborative Research Network. American journal of perinatology. 2018;35(11):1071–8. Epub 2018/04/03. doi: 10.1055/s-0038-1639340 ; PubMed Central PMCID: PMC6436964. 29609190 PMC6436964

[6] Bodnar LM, Parks WT, Perkins K, Pugh SJ, Platt RW, Feghali M, et al. Maternal prepregnancy obesity and cause-specific stillbirth. The American journal of clinical nutrition. 2015;102(4):858–64. Epub 2015/08/28. doi: 10.3945/ajcn.115.112250 ; PubMed Central PMCID: PMC4588742. 26310539 PMC4588742

[7] Flenady V, Koopmans L, Middleton P, Froen JF, Smith GC, Gibbons K, et al. Major risk factors for stillbirth in high-income countries: a systematic review and meta-analysis. Lancet (London, England). 2011;377(9774):1331–40. Epub 2011/04/19. doi: 10.1016/S0140-6736(10)62233-7 . 21496916

[8] Åmark H, Westgren M, Sirotkina M, Hulthén Varli I, Persson M, Papadogiannakis N. Maternal obesity and stillbirth at term; placental pathology-A case control study. PloS one. 2021;16(4):e0250983. Epub 2021/05/01. doi: 10.1371/journal.pone.0250983 ; PubMed Central PMCID: PMC8087010. 33930082 PMC8087010

[9] Åmark H, Sirotkina M, Westgren M, Papadogiannakis N, Persson M. Is obesity in pregnancy associated with signs of chronic fetal hypoxia? Acta obstetricia et gynecologica Scandinavica. 2020;99(12):1649–56. Epub 2020/06/20. doi: 10.1111/aogs.13941 . 32557543

[10] Ford ES. Body mass index, diabetes, and C-reactive protein among U.S. adults. Diabetes care. 1999;22(12):1971–7. Epub 1999/12/10. doi: 10.2337/diacare.22.12.1971 . 10587828

[11] Myatt L, Maloyan A. Obesity and Placental Function. Semin Reprod Med. 2016;34(1):42–9. Epub 2016/01/07. doi: 10.1055/s-0035-1570027 . 26734917

[12] Carbone S, Canada JM, Billingsley HE, Siddiqui MS, Elagizi A, Lavie CJ. Obesity paradox in cardiovascular disease: where do we stand? Vascular health and risk management. 2019;15:89–100. Epub 2019/05/24. doi: 10.2147/VHRM.S168946 ; PubMed Central PMCID: PMC6503652. 31118651 PMC6503652

[13] Varli IH, Petersson K, Bottinga R, Bremme K, Hofsjo A, Holm M, et al. The Stockholm classification of stillbirth. Acta obstetricia et gynecologica Scandinavica. 2008;87(11):1202–12. Epub 2008/10/28. doi: 10.1080/00016340802460271 . 18951207

[14] Åmark H WM. Maternal obesity and stillbirth at term; placental pathology- A case control study. Stockholm: Karolinska Institutet, 2020 [cited 2021 Feb 12]. Report No. 10.1371/journal.pone.0250983 PMC8087010 33930082

[15] Khong TY, Mooney EE, Ariel I, Balmus NC, Boyd TK, Brundler MA, et al. Sampling and Definitions of Placental Lesions: Amsterdam Placental Workshop Group Consensus Statement. Arch Pathol Lab Med. 2016;140(7):698–713. Epub 2016/05/26. doi: 10.5858/arpa.2015-0225-CC . 27223167

[16] Silver RM. Examining the link between placental pathology, growth restriction, and stillbirth. Best practice & research Clinical obstetrics & gynaecology. 2018;49:89–102. Epub 2018/05/16. doi: 10.1016/j.bpobgyn.2018.03.004 . 29759932

[17] Pinar H, Goldenberg RL, Koch MA, Heim-Hall J, Hawkins HK, Shehata B, et al. Placental findings in singleton stillbirths. Obstetrics and gynecology. 2014;123(2 Pt 1):325–36. Epub 2014/01/10. doi: 10.1097/AOG.0000000000000100 ; PubMed Central PMCID: PMC3948332. 24402599 PMC3948332

[18] Kim CJ, Romero R, Chaemsaithong P, Chaiyasit N, Yoon BH, Kim YM. Acute chorioamnionitis and funisitis: definition, pathologic features, and clinical significance. American journal of obstetrics and gynecology. 2015;213(4 Suppl):S29–52. Epub 2015/10/03. doi: 10.1016/j.ajog.2015.08.040 ; PubMed Central PMCID: PMC4774647. 26428501 PMC4774647

[19] Nikkels PG, Evers AC, Schuit E, Brouwers HA, Bruinse HW, Bont L, et al. Placenta Pathology From Term Born Neonates With Normal or Adverse Outcome. Pediatric and developmental pathology: the official journal of the Society for Pediatric Pathology and the Paediatric Pathology Society. 2021;24(2):121–30. Epub 2021/01/21. doi: 10.1177/1093526620980608 . 33470918

[20] Ananthan A, Nanavati R, Sathe P, Balasubramanian H. Placental Findings in Singleton Stillbirths: A Case-control Study. J Trop Pediatr. 2019;65(1):21–8. Epub 2018/02/09. doi: 10.1093/tropej/fmy006 . 29420825

[21] Roberts KA, Riley SC, Reynolds RM, Barr S, Evans M, Statham A, et al. Placental structure and inflammation in pregnancies associated with obesity. Placenta. 2011;32(3):247–54. Epub 2011/01/15. doi: 10.1016/j.placenta.2010.12.023 . 21232790

[22] Goldstein JA, Gallagher K, Beck C, Kumar R, Gernand AD. Maternal-Fetal Inflammation in the Placenta and the Developmental Origins of Health and Disease. Frontiers in immunology. 2020;11:531543. Epub 2020/12/08. doi: 10.3389/fimmu.2020.531543 ; PubMed Central PMCID: PMC7691234. 33281808 PMC7691234

[23] Olson KN, Redman LM, Sones JL. Obesity "complements" preeclampsia. Physiological genomics. 2019;51(3):73–6. Epub 2019/02/05. doi: 10.1152/physiolgenomics.00102.2018 ; PubMed Central PMCID: PMC6459374. 30716010 PMC6459374

[24] Kawai T, Autieri MV, Scalia R. Adipose tissue inflammation and metabolic dysfunction in obesity. American journal of physiology Cell physiology. 2021;320(3):C375–c91. Epub 2020/12/29. doi: 10.1152/ajpcell.00379.2020 ; PubMed Central PMCID: PMC8294624. 33356944 PMC8294624

[25] Lopez-Jaramillo P, Barajas J, Rueda-Quijano SM, Lopez-Lopez C, Felix C. Obesity and Preeclampsia: Common Pathophysiological Mechanisms. Frontiers in physiology. 2018;9:1838. Epub 2019/01/09. doi: 10.3389/fphys.2018.01838 ; PubMed Central PMCID: PMC6305943. 30618843 PMC6305943

[26] Ouchi N, Parker JL, Lugus JJ, Walsh K. Adipokines in inflammation and metabolic disease. Nature reviews Immunology. 2011;11(2):85–97. Epub 2011/01/22. doi: 10.1038/nri2921 ; PubMed Central PMCID: PMC3518031. 21252989 PMC3518031

[27] Amodeo S, Cavoretto PI, Seidenari A, Paci G, Germano C, Monari F, et al. Second trimester uterine arteries pulsatility index is a function of placental pathology and provides insights on stillbirth aetiology: A multicenter matched case-control study. Placenta. 2022;121:7–13. Epub 2022/03/05. doi: 10.1016/j.placenta.2022.02.021 . 35245721

[28] Chiofalo B, Laganà AS, Vaiarelli A, La Rosa VL, Rossetti D, Palmara V, et al. Do miRNAs Play a Role in Fetal Growth Restriction? A Fresh Look to a Busy Corner. BioMed research international. 2017;2017:6073167. Epub 2017/05/04. doi: 10.1155/2017/6073167 ; PubMed Central PMCID: PMC5390605. 28466013 PMC5390605

[29] Socialstyrelsen. https://www.socialstyrelsen.se/SiteCollectionDocuments/metodbeskrivning-kunskapsunderlag-graviditetsdiabetes-remissversion.pdf.

[30] Socialstyrelsen. Statistikdatabas för graviditeter, förlossningar och nyfödda [Internet]. Stockholm: Socialstyrelsen; 2021. Available from: https://sdb.socialstyrelsen.se/if_mfr_004/resultat.aspx.

